# Evaluation of the ulna lengthening by distraction osteogenesis in congenital radial deficiency

**DOI:** 10.1007/s00590-022-03381-1

**Published:** 2022-09-06

**Authors:** Michał Górecki, Marcin Redman, Leszek Romanowski, Piotr Czarnecki

**Affiliations:** 1 Department of Traumatology, Orthopaedics and Hand Surgery, Poznań University of Medical Sciences, ul. 28 Czerwca 1956 r. nr 135/147, 61-545 Poznań, Poland; 2 Wiktor Dega Orthopedic - Rehabilitation Clinical Hospital, Poznań University of Medical Sciences, Poznań, Poland

**Keywords:** Radial longitudinal deficiency, Radial club hand, Congenital disorders, Ulna lengthening, Ulna elongation, Distraction osteogenesis

## Abstract

**Purpose:**

Publications evaluating the results of the ulna lengthening in congenital radial deficiency are based only on small groups of subjects which yield statistical studies of low scientific value. The aim was to examine the effectiveness of ulna lengthening in radial longitudinal deficiency and determine the number and quality of complications based on one of the most numerous study groups described in the literature.

**Methods:**

The material consists of a study group with 31 upper limbs of unmatured patients diagnosed with type III and IV radial longitudinal deficiency. The study group was evaluated based on the parameters known from the literature. The difficulties during elongation were classified according to Paley’s classification.

**Results:**

The study group contained patients with a mean age of 9 years, and the number of boys and girls was comparable. Ulna length significantly increased after elongation compared to the initial bone length. The patient’s age didn’t affect the ulna lengthening, and the amount of elongation didn’t significantly affect the total stabilization period. However, the total stabilization time increased with increasing patient age. Difficulties affected more than half of the cases.

**Conclusions:**

Ulna elongation in congenital radial deficiency results in significant lengthening of the ulna, and thus the entire forearm, compared to the initial bone length. This technique has a high percentage of difficulty, so its use should be considered after cautious discussion with the parents and patients.

## Introduction

Congenital radial deficiency is generalized underdevelopment of the upper extremity with shortening and bending of the forearm and radial deviation and displacement of the wrist about the distal end of the ulna. There is always underdevelopment of the thumb in a wide range of severity, from slight hypoplasia to complete aplasia. The incidence ranges from 1 in 30,000 to 1 in 100,000 live births. This defect occurs in 38–66% bilaterally and often asymmetrically. It may be a component of congenital anomalies such as Holt-Oram syndrome, VACTERL, or TAR or co-occur with other congenital abnormalities such as Fanconi's anemia or syndactyly. Most cases occur spontaneously without being inherited [1–5].

The most common classification of congenital radial deficiency is the four-stage classification proposed by Bayne and Klug based on an X-ray evaluation of the forearm bones [6].

Treatments include conservative procedures, which are reserved for patients with slight deformity and stable joints [1, 3, 7], and surgery, where centralization [1, 5, 8, 9], radialization [10], or ulnarization [11] of the wrist at the distal end of the ulna is the standard procedures. Additional surgical procedures may include osteotomy and distraction osteogenesis of the bent ulna, which improve the aesthetic appearance and reduce the length of the forearm relative to a healthy extremity [12]. In addition, depending on the severity of thumb underdevelopment, reconstructive surgeries are performed, such as stabilization of the first metacarpophalangeal joint, deepening of the first web space, relocation of the interphalangeal joint from the foot to the first carpometacarpal joint, and finally amputation with index finger pollicization [1, 13–15].

Publications evaluating the results of the ulna lengthening in congenital radius deficiency are based only on small groups of subjects. The resulting statistical studies are of low scientific value [16–19].

This study aims to examine the effectiveness of ulna lengthening in congenital radial deficiency.

Also, we determine the number and type of difficulties encountered during distraction osteogenesis.

## Material

The study group contains 31 upper extremities in 28 patients (12 females, 16 males) who underwent ulna lengthening once by single distraction osteogenesis. All patients were diagnosed with type III and IV congenital radial deficiency, according to the Bayne and Klug classification [6]. In 25 cases, the defect was unilateral, while in three cases, it was bilateral. Five patients were additionally diagnosed with congenital syndromes: Holt–Oram syndrome (2 patients), Nager syndrome (1 patient), TAR syndrome (1 patient), and Klippel–Feil syndrome (1 patient).

The study is retrospective and based on clinical data and X-rays of patients treated in the hand surgery clinic until 2020. Patients before reaching 18 years of age were evaluated.

## Method

### Operating technique

**Fig. 1:**
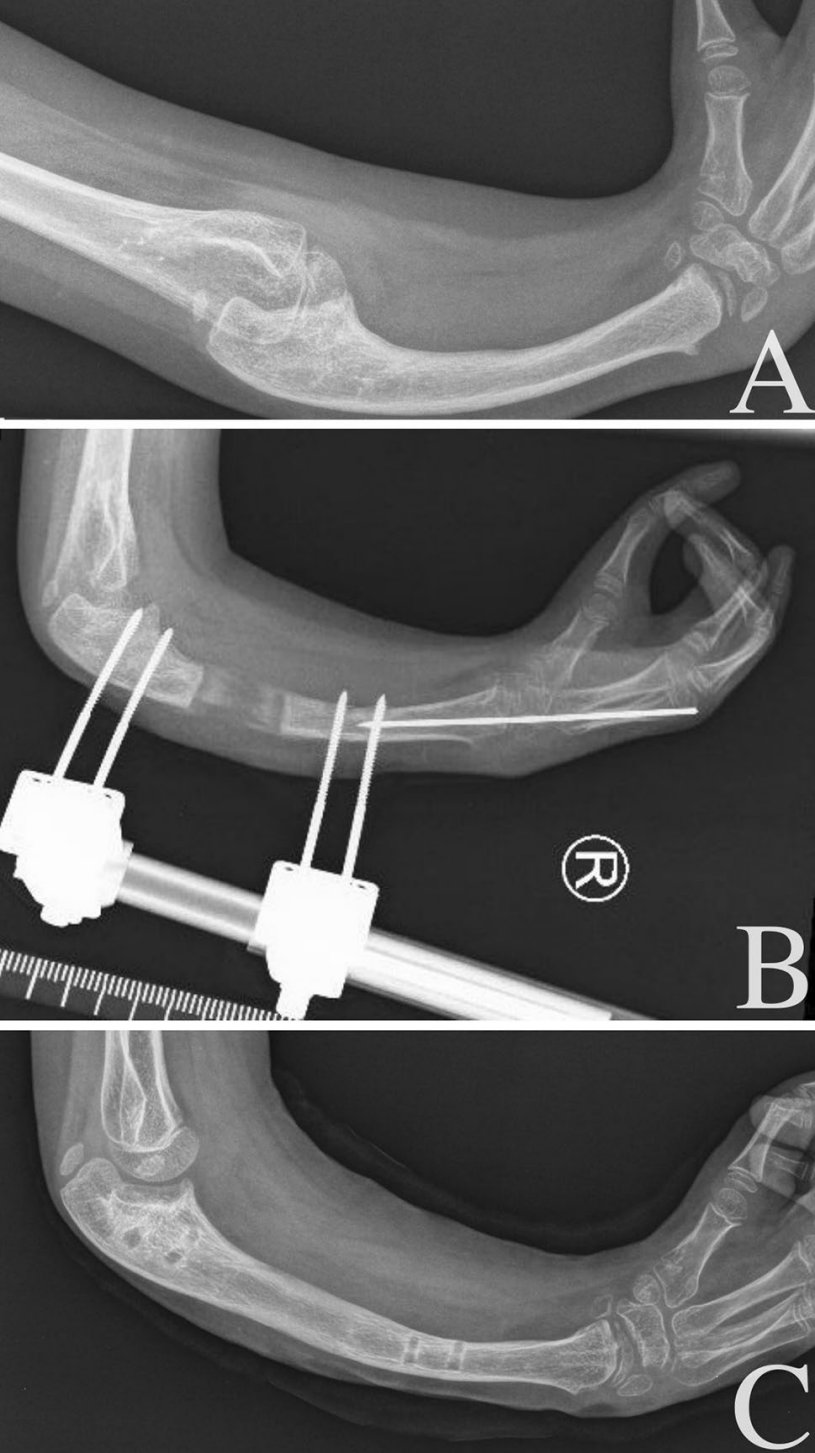
X-ray of the ulna before (**a**), during (**b**), and after (**c**) lengthening

The ulna lengthening procedure has been used in hand surgery clinic since the 1990s. This technique is based on the assumptions proposed by Ilizarov regarding distraction osteogenesis. The method has undergone only minor modifications over the years. The PUMED monolateral external osteogenesis distractor is used, the osteotomy is performed subperiosteally, and the distraction rate is 4 × ¼ mm per day, with possible correction while lengthening. (Fig. 1).

### Parameters

The following parameters, known from the literature, were evaluated based on clinical data and radiographs:

- Initial length [mm]—ulna length before lengthening
- Final length [mm]—ulna length after lengthening
- Lengthening [mm] = final length—initial length
- % of length increase = lengthening / initial length × 100%—this parameter defines what percentage of the original length of the examined bone is the achieved lengthening
- Total stabilization period [month]—the time between insertion and removal of the distraction device
- Healing rate [month/cm] = (total period of stabilization / 30) / lengthening in cm—this parameter describes how much time in months is needed to achieve bone lengthening by 1 cm.

### Measurement

The ulnas were evaluated using properly taken radiographs in the lateral projection. The study group was assessed before and after bone lengthening, and the radiographs were taken up to 7 days before both insertion and removal of the distraction device. The bone lengths were measured as described in Heikel’s 1959 paper, determining functional bone length rather than actual [20]. He proposed the following lines on the X-ray.

**Fig. 2:**
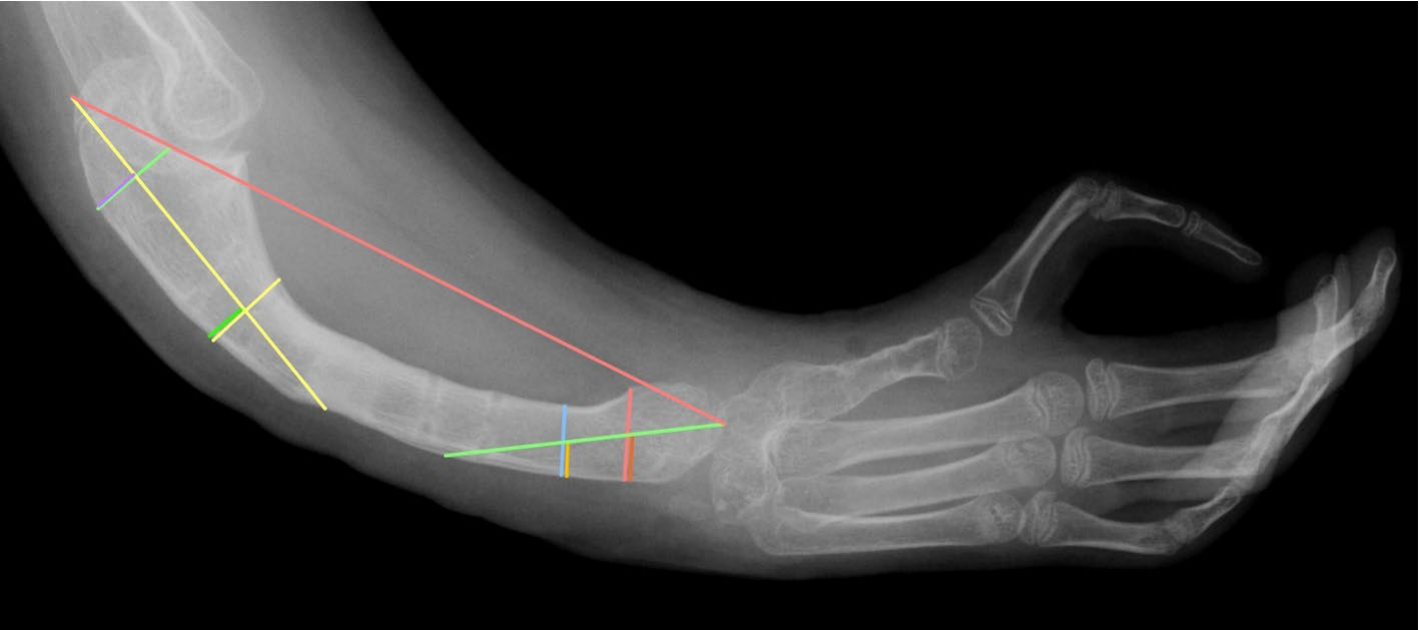
The method of measuring the ulna length proposed by Heikel

Two straight lines between the outer contours of the cortical layers of the proximal end of the ulna, the first at the lowest point of the trochlear notch, the second anteriorly from the coronoid process. The axis of the proximal end of the ulna runs through the centers of these lines. In the same way, Heikel traced two lines in the distal end, the locations he chose randomly. The centers of these lines mark the axis of the distal end. The functional ulna length was defined by the segment between the points of intersection of the proximal ulna axis with its proximal end and the axis of the distal ulna part with its distal end. (Fig. 2). The radiographs were evaluated by using a specialized computer program.﻿

### Evaluation of the difficulties encountered during lengthening

The difficulties that occurred during the total stabilization period of limb lengthening were divided, according to the classification proposed by Paley in 1990 [21], into:

- Problems—difficulties removed nonoperatively before completion of lengthening, e.g., axis correction
- Obstacles—difficulties requiring surgical intervention to remove them, e.g., premature bone union
- Complications—intraoperative difficulties and problems not solvable before the completion of lengthening, e.g., pseudarthrosis

### Statistical analysis

The obtained results were statistically analyzed using MedCalc Statistical Software, version 19.6.0.0. Statistical tests were appropriately selected depending on the scale on which the analyzed variables were described, the nature of the distribution of the results, and their possible correlations. The existence of differences between significant variables within the study group was examined. The presence of possible correlations between the variables was also determined.

Quantitative characteristics were described by the mean and standard deviation (SD). The confidence interval at which a result was considered statistically significant was adopted for *p* < 0.05.

## Results

The study group contained 31 upper limbs with a congenital radial deficiency. The mean age of the patient was nine years, and the number of boys and girls was comparable.

After distraction osteogenesis for an average of 5 months, the lengthening of approximately 20% was achieved, resulting in a significant increase in length compared to the initial bone length. This gives a healing rate of 2.7 months/centimeter (Table 1).

**Table 1:** Table summarizing the descriptive statistics of the study group and the *p*-value of comparisons of selected variables

	Study group	*p*-value
Number of the ulnas	31	
Gender (m/f)	14/17	
Age [years]	9,4 ± 3,4	
Initial length [mm]	117,1 ± 28,9	*P* < 0,05
Final length [mm]	139,8 ± 32,1	
Lengthening [mm]	23 ± 9	
Length increase [%]	20 ± 8	
Stabilization period [months]	5 ± 2	
Healing rate [months/cm]	2,7	

**Fig. 3:**
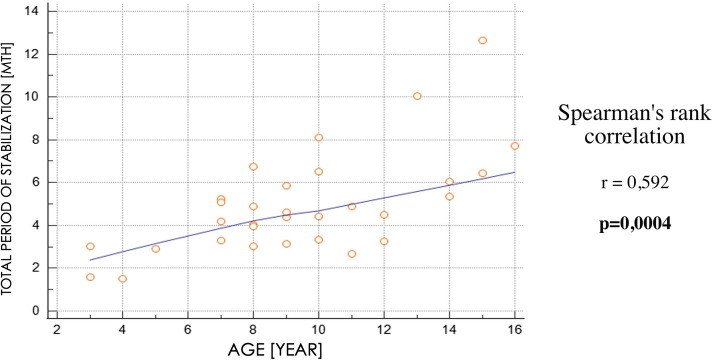
Spearman's rank correlation between the total period of stabilization and patient’s age

The patient’s age did not significantly affect the degree of ulna lengthening, and the amount of elongation did not significantly affect the total stabilization period. However, the entire stabilization period increased significantly with increasing patient age (Table 2; Fig. 3). Difficulties encountered during distraction osteogenesis affected more than half of the lengthened ulnas, and the most common were obstructions (Table 3).

**Table 2:** Spearman's rank correlation coefficient between selected parameters of the study group

	Elongation [MM]	Increase in length [%]	Stabilization period [months]
Age [Years]	0,226	− 0,143	0,592*
stabilization period [months]	0,339	− 0,020	

*p < 0,05

**Table 3:** Difficulties encountered during the total stabilization period of limb lengthening according to the Paley classification

Difficulties during lengthening	
Problems	3
Obstacles	8
Complications	6
	17/31 (55%)

## Discussion

Shortening of the forearm that occurs in congenital radial deficiency is a significant clinical and aesthetic problem for the patient.

The publication includes patients with type III and IV congenital radial deficiency, with significant forearm axis shortening and bending. In Type I and II, shortening is minimal, and ulnar lengthening is not used as a treatment option [6].

The natural growth of the ulna during congenital radial deficiency type III and IV is estimated at 50–75% of the standard bone length in the literature. This ratio remains constant throughout the growth period [3, 17, 20, 22]. This is a significant shortening, which should be treated surgically. In addition to surgeries leading to forearm lengthening [23], other surgeries are performed to correct wrist alignments, such as centralization or ulnarization, and reconstructive surgeries related to thumb hypoplasia/aplasia. Moreover, Glossop et al. [24] proved mathematically that correction of the ulna bend would not cause its significant elongation, even in the case of multiple opening osteotomies. Because of the above, numerous attempts to lengthen the ulna seem most justified.

The study group of 31 ulnar lengthening in congenital radial deficiency is the largest in the available literature. The results obtained differ from the literature based on smaller study groups [16–19]. The findings of our study show ulna lengthening of an average of 23 ± 9 mm, representing an average increase in original bone length of 20 ± 8%. The overall stabilization period averaged 5 months (20 weeks) with an average healing rate of 2.7 months/cm (10.8 weeks/cm).

In their study, Pickford et al. [17] evaluated the lengthening of eight ulnas in patients with an average age of 10 years using the Ilizarov appliance. They obtained a mean bone lengthening of 4.7 cm, which was 46% of the original length. The healing rate was 3.8 weeks/cm. The authors did not provide information on the total period of stabilization but reported a mean lengthening time of 15 weeks and suggested that stabilization be maintained more than 4 weeks after lengthening to achieve satisfactory consolidation. Difficulties during lengthening occurred in all patients, including implant infections, night pain, regenerative fractures, and delayed union.

Similar values of ulna lengthening are presented in their study by Peterson et al. [16]. Based on 13 cases of lengthening, they achieved an average bone growth of 4.4 cm, but they do not present what percentage of the original bone length this is. Lengthening took an average of 14.4 weeks, with an average consolidation time of 23 weeks. These data can determine the healing rate, which averaged 8.6 weeks/cm. During lengthening, complications in the form of infection around the implants affected all patients, elbow and finger stiffness affected 60% of cases, and 40% were diagnosed with missing or defective union requiring surgical intervention.

There is a significantly lower healing rate in the work of Pickford et al. compared to the work of Peterson et al. This indicates a much shorter total period of stabilization of the lengthened bones, which affects the shorter consolidation time of the regenerate, and thus may be the reason for the higher rate of regenerate fracture and delayed bone union present in 50% of cases.

In their publication, Raimondo et al.[18] evaluated the lengthening of the four ulnas obtaining a result of 6.1–8.1 cm. At the same time, they also did not report what percentage of the original bone length it was. The authors obtained these values at the expense of a long period of complete stabilization of 8–10 months, so the healing rate was 1.2–1.6 months/cm (4.8–6.4 weeks/cm). This degree of ulna lengthening in most patients was associated with difficulties in the form of finger contractures and, in one case, wrist and elbow contractures.

Another aim of this study was to evaluate the number and type of difficulties encountered during ulnar lengthening. We used the classification proposed by Paley, who defines it as difficulty and divides it into problems, obstacles, and complications [21]. In our study group, difficulties occurred in 55% of the extremities being lengthened. According to the proposed classification, we can distinguish 18% of problems, 47% of obstacles, and 35% of complications. Paley’s classification has been used for many years and is intended to allow the comparison of results between authors of works. Unfortunately, it is much more used in papers describing lower extremity lengthening, and most cases of complications associated with upper extremity lengthening remain unclassified.

One of the significant doubts about the elongation of the ulna in a child with congenital radial deficiency of type III and IV would be if it ultimately results in a real length gain or whether the child's growth would result in a loss of the length gain achieved in lengthening. Lengthening is suspected to increase the forces acting on the tissues and may cause a decrease in length by disrupting the growth cartilage region.

The mechanism by which ulna growth is impaired is still unknown and lengthening by distraction osteogenesis improves length only to some extent. It is important to remember that this technique carries a challenging number of complications, so its use must be considered with extreme caution [3]. Frequent patient monitoring can minimize the difficulties that occur during the lengthening process. In addition to the physical evaluation, additional examinations such as X-rays and ultrasound are crucial and may help adjust the lengthening rate to the condition of the forming regenerate. Tetsworth et al. suggest that the gap between the ends of the forming regenerate should not be greater than 5 mm on an X-ray. Exceeding the recommended value may indicate a faster distraction process than bone formation. In this case, compression of the ends of the regenerate should be considered for a few days to achieve consolidation, and then lengthening should be resumed at a slower rate [25]. A more accurate way of monitoring is to assess bone formation by ultrasound, a simple and widely available examination [26].

When lengthening the ulna, the effect of months of treatment on the child’s development should also be considered. The age at which to undertake this debilitating treatment for both patient and parents is a consideration. In the most unilateral congenital radial deficiency of type III and IV, the ulna lengthening does not achieve equal limb length. For this reason, multiple elongations within the same bone are attempted to sum up to achieve a more significant length gain. However, Yoshida et al. [27] point out in their work that complications are more frequent and more severe when the same bone segment is lengthened again. In addition, it is essential to remember that, according to Catagni et al. [28], excessive forearm lengthening often increases finger stiffness, thus decreasing overall hand function.

It is also essential to know the effect of bone elongation on subsequent bone growth. It appears that the results are inconclusive, and the topic is controversial among the authors. McCarthy et al. [29] conducted such a study in the lower extremities, where an average of 24% bone lengthening showed no statistically significant differences between the growth rate of limbs after lengthening and the growth rate of non-lengthened limbs. Hope and Sabhaewal [30, 31], among others, present similar data in their work, while some authors like Sharma and Viehweger [32, 33] note a significant slowing of bone growth rate after bone lengthening. Lee et al. [34] evaluated the behavior of bone epiphyses after lengthening using an animal model. They noted that a 20% lengthening does not cause significant changes in the epiphyses, regardless of the distraction rate. Only lengthening by more than 30% results in a significant decrease in the growth rate of the lengthened bone, and exceeding 50% results in premature closure of the growth cartilage.

The doubts mentioned above could be answered by a study with a control group, which would evaluate the length of the ulna in patients with congenital radial deficiency of type III and IV but without ulna lengthening (as a natural course of the disease) and in patients after ulna lengthening (study group) with re-measurement after the period of bone growth. The resulting data could assess whether the growth of an elongated ulna would be similar or reduced compared to its natural growth.

Publications evaluating the results of the ulna lengthening in congenital radius deficiency are based only on small groups of subjects. The resulting statistical studies are of low scientific value. Our cohort study is based on one of the most numerous study groups described in the literature. We observed a significantly greater ulnar final bone length in the group of patients where single bone elongation was used as one of the treatment methods. We achieved an average of 20% elongation of the ulna. In addition, we observed that the degree of bone lengthening was similar regardless of patient age, whereas the total stabilization time increased with age. This suggests that as the patient ages, the need to increase the entire stabilization period increases to achieve a similar bone lengthening effect as in younger patients. In our study group, 55% of the extremities lengthened difficulties were present.

### The limitations of the study

1. The ulna lengthening in congenital radial deficiency may improve limb function. The question remains, what percentage of elongation significantly improves its function? This should be analyzed/studied in future research papers.
2. The study does not evaluate the effect of ulna lengthening on its natural growth after elongation. It would be beneficial to assess the length of the ulna again after the period of bone growth and add the control group of patients with the defect mentioned above without ulna elongation.
3. Although the study group was probably the largest in the available literature; still, the number of patients studied could be more significant for a more accurate statistical analysis.
4. The parameters evaluated: degree of lengthening, total stabilization period, and healing rate depended on the decisions made during the therapeutic process, which often proceeded with difficulty.

## Conclusions

Distraction osteogenesis of the ulna performed in types III and IV of congenital radial deficiency results in significant lengthening of the ulna, and thus the entire forearm, compared to the initial bone length. This procedure, combined with other surgical interventions such as wrist centralization or corrective osteotomy of the ulna, can improve the affected extremity's function and correct a less important aesthetic problem.

This technique has a high percentage of difficulty, so its use should be considered cautiously after a thorough discussion with the parents and/or patients.
